# Attachment and Emotional Eating: A Scoping Review Uncovering Relational Roots to Inform Preventive Healthcare

**DOI:** 10.3390/healthcare13233170

**Published:** 2025-12-04

**Authors:** Pamela Nader, Hilda E. Ghadieh, Nivine Abbas, Nayla Nahas

**Affiliations:** 1 Department of Psychology, Faculty of Arts and Sciences, University of Balamand, El Koura, Tripoli 100, Lebanon; 2 Department of Biomedical Sciences, Faculty of Medicine and Medical Sciences, University of Balamand, El Koura, Tripoli 100, Lebanon; hilda.ghadieh@balamand.edu.lb; 3 Public Health Department, Faculty of Health Sciences, University of Balamand, El Koura, Tripoli 100, Lebanon; nivine.abbas@balamand.edu.lb

**Keywords:** attachment representation, attachment style, eating behavior, emotional eating, social determinants of health

## Abstract

**Highlights:**

- Secure attachment styles serve as a shield against emotional eating, while anxious attachment heightens vulnerability.
- Stress acts as a key moderator, shaping how attachment influences emotional eating.
- Emotion regulation and body awareness bridge the link between attachment and emotional eating.
- Research rarely delves into early attachment representations and child–caregiver bonds.
- Findings remain culturally narrow, with limited diversity in age, gender, and global representation.

**What are the main findings?**

- Attachment insecurity, particularly attachment anxiety, is consistently associated with greater emotional eating, whereas secure attachment is generally protective. This relationship is evident across general attachment styles and, in adults, specific attachment figures (e.g., romantic partners).
- Psychological mechanisms such as emotion regulation difficulties, perceived hunger, body dissatisfaction, and stress moderate or mediate the attachment–emotional eating link, highlighting the complexity and context dependence of this relationship.
- The review calls for research on diverse populations, gender, and pubertal factors, psychological mediators, and caregiver attachment links to emotional eating.

**What are the implications of the main findings?**

- Integrating attachment- and emotion-focused strategies in preventive healthcare may reduce emotional eating. Considering body dissatisfaction, perceived hunger, stress, and cultural or developmental context can improve the design of behavioral and nutritional programs to prevent emotional eating.

**Abstract:**

**Background/Objectives**: Emotional eating can pose a significant threat to one’s health as it can be a predictor of eating disorders. It involves eating in response to emotional distress rather than physical hunger and is widely associated with insecure attachment patterns. However, it remains unclear whether this relationship is consistent across cultures and in the general population. This review aimed to examine the relationship between different conceptualizations of attachment and emotional eating in non-clinical adolescent and adult populations. **Methods**: Nineteen eligible studies were identified through searches of five databases, including ProQuest, Scopus, Google Scholar, PubMed, and ProQuest Dissertations and Theses Global (PQDT), which covers peer-reviewed literature published between 1990 and 2025. Both quantitative and qualitative studies were included, spanning attachment styles, representations, and states. **Results**: The findings consistently revealed that general secure attachment styles are associated with lower levels of EE. In comparison, insecure–anxious and disorganized attachment styles are positively associated with higher EE levels. In contrast, avoidant attachment showed a weak or inconsistent association. Mediators such as difficulties with emotion regulation, perceived hunger, and body dissatisfaction were frequently identified, and stress emerged as a relevant moderator. Notably, attachment to specific figures (e.g., romantic partners versus caregivers) and cultural context were poorly addressed, which may influence the strength and consistency of the associations. The review also highlights conceptual gaps in the literature, including a limited focus on early attachment representations, context-specific attachment scripts, and the cultural validity of these concepts. Most studies were cross-sectional and conducted in Western contexts, which limited their causal interpretation and generalizability. **Conclusions**: These findings underscore the importance of attachment-based frameworks in understanding and preventing EE. They suggest the necessity to conduct further studies that are more nuanced, longitudinal, culturally diverse, and that consider sex and pubertal changes for a better understanding of the relationship between attachment and EE. This review contributes to prevention efforts and global health strategies by addressing the relational and emotional determinants of unhealthy eating behaviors in the general population.

## 1. Introduction

Eating is tied to emotions, self-perception, and social contexts, and often serves social functions. However, using food to manage emotions can backfire, leading to unhealthy eating habits and potential obesity [1]. Chew et al. suggest that emotional eating is significantly prevalent at 44.9% in overweight and obese populations, which makes it a significant risk factor for health issues [2]. Emotional eating (EE) is typically defined as eating in response to negative emotions [3]; however, some individuals may also eat in response to positive emotions [4]. It often reflects a need to numb emotional pain and replace human connection [5].

While stress may suppress appetite, it can also increase food intake due to elevated cortisol and insulin levels, which contribute to metabolic issues [6,7]. Emotional eating, in both its forms, i.e., emotional overeating (EO) and emotional undereating (EU) [8] is driven by negative psychological states such as guilt [9], anxiety, depression, anger/frustration [10], and is linked to pleasure seeking rather than nutrition, with the preferred foods being mainly energy-dense, poor in nutrients, and tasty [11,12]. This is particularly critical in cases where emotional eating triggered by negative emotions contributes to increased BMI, thereby elevating the risk of obesity-related health complications [13].

According to Bowlby’s attachment theory, the child–caregiver bond is conceptualized as a system of interaction between the child and the caregiver [14]. It describes how internal and external stressors form a motivational system that drives infants to seek proximity to a significant caregiver. A comforting caregiver thus becomes a secure haven and a secure base, activating the child’s system of exploring the environment. Early repetitive patterns of caregiver-child interactions are mentalized as Internal Working Models, cognitive frameworks that shape interactions between self and others and further influence relationships throughout life [14]. The interplay of these attachment and exploratory behaviors, as observed by Mary Ainsworth [15] and Mary Main and Georges Solomon [16], yielded four behavioral patterns in seeking security and became the basic prototypes of attachment categories: secure, anxious/ambivalent, avoidant/disorganized. Attachment has been conceptualized through multiple constructs, each reflecting distinct psychological processes. These include *attachment behaviors* (observable proximity-seeking and separation protest behaviors assessed through tools like the Strange Situation Procedure (SSP) [15] and the Attachment Q-Set (AQS) [17]), *attachment representations* (cognitive schemas of early caregiver relationships measured via interviews such as the Adult Attachment Interview (AAI) [18], or self-report such as the CaMIR [19]), and *attachment styles* (self-perceptions of general relational patterns [20] assessed by instruments such as the Relationship Questionnaire (RQ) [21] and the the Revised Adult Attachment Scale (RAAS) [22]; or specific relational patterns assessed by the Experiences in Close Relationships scale (ECR) [23]). More recently, state attachment has been introduced to capture context-dependent activation of attachment scripts [24] using tools like the SAAM [25]. Assessments differ not only in the attachment figure targeted—general bonding tendencies versus relationships to specific attachment figures—but also in approach, with both categorical (secure, anxious, avoidant, disorganized) and dimensional models (continuums of anxiety and avoidance) employed. While categorical measures offer clinical utility, dimensional scales dominate research due to their statistical flexibility, typically operationalizing security as low anxiety and avoidance [21].

Because feeding and the alleviation of hunger-related discomfort play a central role in the early regulation of child–caregiver interactions, attachment theory has become a relevant and valuable framework for understanding the development of eating behaviors, particularly emotional eating. Several studies have explored the relationship between insecure attachment and EE, considering various mediators and moderators that might influence this connection. Potential mediating factors, such as emotion suppression [26], alexithymia [26], rumination [27], difficulties in emotion regulation [28], body dissatisfaction [29], and interoceptive awareness [30], have been identified in the literature. However, it remains unclear if their role is consistent across different studies. Additionally, although most cited research focuses on populations from Western socio-cultural contexts, it is unclear whether the same findings hold across countries.

Moreover, while previous reviews have focused mainly on attachment in clinical populations with diagnosed eating disorders [31], few have synthesized data from non-clinical samples where early identification and prevention are still possible. To our knowledge, only one review [32] has been conducted on the general population. This meta-analysis examined the link between attachment and a broad spectrum of unhealthy eating behaviors—including binge eating, bulimic symptoms, dieting, emotional eating, and unhealthy food consumption—in the general population. However, by grouping pathological and non-pathological behaviors, it became challenging to isolate the specific association between attachment and emotional eating (EE), which, while non-pathological, is highly pathogenic. Furthermore, although Faber [32] explored categorical versus dimensional approaches to measuring attachment, the review did not consider the conceptual heterogeneity of attachment in relation to emotional eating research. Different attachment constructs and measurement approaches may tap into distinct psychological mechanisms that influence eating behaviors in unique ways. Without clarifying these distinctions, findings across studies remain difficult to compare, and theoretical models risk oversimplification. Our review addresses this gap by synthesizing evidence across diverse frameworks to elucidate the relationship between specific attachment processes and emotional eating, as well as the mechanisms underlying this relationship. Finally, unlike Faber’s review, we incorporate cultural variability, adding an essential dimension to understanding how socio-cultural contexts shape these associations. This review has the following objectives:

1. Explore whether attachment, as a style or as a representation, is associated with emotional eating in the general population of adolescents and adults, and if this association exists in different socio-cultural contexts.
2. Explore the mechanisms that mediate and moderate the relationship between attachment and emotional eating and their relevance to different socio-cultural contexts.

By synthesizing the literature and identifying gaps—particularly in cultural differences, mediators, and generalizability—this review may support future interventions and inform public health strategies. Ultimately, it aligns with Sustainable Development Goal 3 (SDG 3): Good Health and Well-being [33] by addressing the psychological and relational determinants of unhealthy eating and contributing to population-level prevention efforts.

## 2. Materials and Methods

### 2.1. Search Strategy

This review was completed following a systematic search for articles that included the following terms: “emotional eating,” “eating behavior,” “stress eating,” “comfort eating,” and “attachment,” combined using Boolean operators. A comprehensive search strategy was developed, utilizing major electronic databases, including ProQuest (2024, Clarivate, Ann Arbor, MI, USA, https://www.proquest.com), Scopus (Elsevier, 2024, Amesterdam, Netherlands, https://www.scopus.com), Google Scholar (2024 Google LLC, Mountain view, CA, USA, https://scholar.google.com), and PubMed (2024, National Library of Medicine, Bethesda, MD, USA, https://pubmed.ncbi.nlm.nih.gov), as well as grey literature from ProQuest Dissertationsand Theses Global (2024, Clarivate, Ann Arbor, MI, USA, ProQuest Dissertations & Theses Global (PQDT): https://www.proquest.com/products-services/dissertations/). Searches were restricted to peer-reviewed articles and theses in English published between 1 January 1990 and 31 May 2025.

### 2.2. Eligibility Criteria

The following studies were included in this review:

Studies that had to measure attachment towards a significant other as conceptualized by Bowlby’s theory in terms of attachment patterns, attachment styles, attachment representations, and attachment states were included.

We also included studies that involved measuring emotional eating, defined as eating in response to emotions rather than physical hunger. Situational disinhibited eating will not be included as it is occasional and does not qualify as an individual regulatory pattern.

Studies that had to target only non-clinical adolescent and adult samples were also included. The adolescent period, from the onset of pubertal changes to young adulthood, is a critical time for understanding both healthy and disordered eating [34,35]. It is a critical period where emotional eating often first occurs and becomes more pronounced [36] and is linked with mood swings and emotional dysregulation [37]. Additionally, adulthood is when long-term patterns are often established.

Quantitative, qualitative, or mixed-methods studies were included, allowing for a rich exploration of participants’ experiences that might not be captured by quantitative methods alone.

Excluded from this review were studies that met the following criteria:

Targeted population with eating disorders;

Included clinical samples;

Did not align with established attachment constructs;

Lacked a specific measure of emotional eating;

Treated emotional eating as a mediator of other variables.

Although the language of the study was not a criterion for inclusion or exclusion, all studies found were written in English, probably because it is the most common language used in peer-reviewed journals [38].

### 2.3. Screening, Selection, and Data Extraction

**Figure 1:**
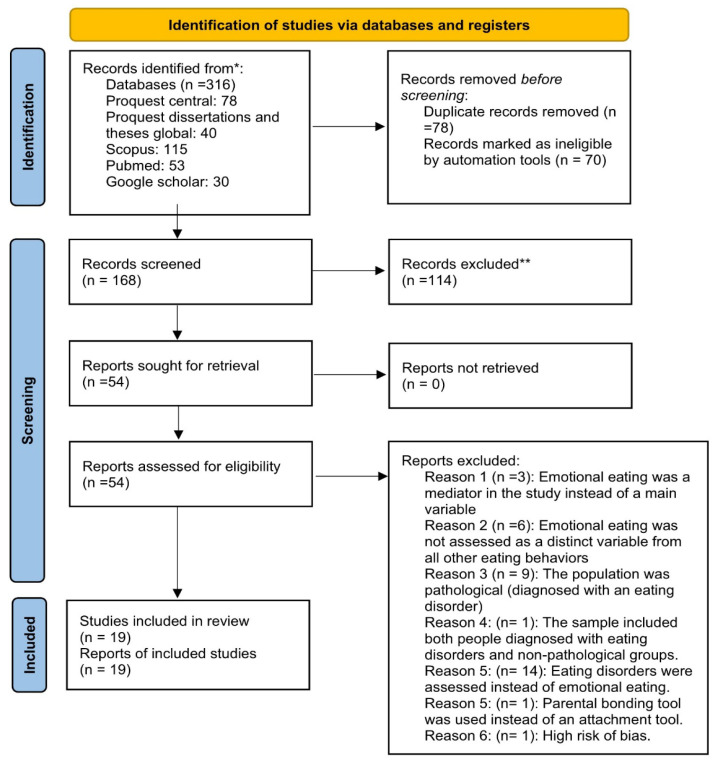
Flow diagram: * and ** studies identification and selection.

The search yielded a total of 316 records, distributed as follows: 78 from ProQuest Central, 40 from ProQuest Dissertations and Theses Global, 115 from Scopus, 53 from PubMed, and 30 from Google Scholar. These results were imported into RefWorks, where 78 duplicates were removed from the 316 identified records. Additionally, the Rayyan application [39] was employed to facilitate a more efficient screening process, particularly by handling the large volume of records and enabling blinded, independent screening. As a result, 70 records were deemed ineligible and removed from further analysis. Two reviewers—the first author and the corresponding/last author—independently screened the remaining 168 records for relevance based on their titles and abstracts, and then based on the full text. Discrepancies were resolved by consensus. 114 records were excluded during the initial screening phase because they were irrelevant to the research question or failed to meet the inclusion criteria. For full-text retrieval, 54 reports were sought. 31 were excluded for the following reasons: emotional eating was treated as a mediator rather than a primary variable (n = 3), emotional eating was not assessed as a distinct variable from other eating behaviors (n = 6), the study population was clinical, involving individuals diagnosed with an eating disorder (n = 9), the sample included both individuals with eating disorders and non-clinical groups (n = 1), eating disorders were assessed instead of emotional eating (n = 14), or parental bonding tool was used instead of an attachment tool (n= 1), high risk of bias (n = 1). Ultimately, 19 studies met the inclusion criteria and were included in this review. The flow diagram is shown in Figure 1.

Although not mandatory for scoping reviews, we conducted a preliminary risk-of-bias assessment to include only studies with low or moderate risk, thereby enhancing the rigor and credibility of our synthesis. Risk of bias was assessed independently by two reviewers using design-specific tools: AXIS (cross-sectional), Newcastle–Ottawa (cohort), RoB 2 (RCTs), and CASP (qualitative). Discrepancies were resolved by consensus (Table S1 in the Supplementary Materials).

We developed a comprehensive data extraction sheet to record systematically, for each article, the following elements: (a) the attachment constructs assessed, (b) the instruments employed for their measurement, and (c) the specific attachment figure targeted, when applicable. The sheet also captured details on emotional eating (EE) assessment tools, as well as mediating and moderating mechanisms through which attachment may influence EE, when reported. Finally, statistical findings were included. The extraction sheet was piloted before implementation and subsequently applied to all eligible studies. Data extraction was conducted independently by two researchers—the first author and the corresponding/last author—with discrepancies resolved through discussion and consensus to ensure accuracy and consistency. For a detailed overview of study characteristics and measurement approaches, refer to Table S2 (quantitative studies with adolescents), Table S3 (quantitative studies with adults), and Table S4 (qualitative studies with adults).

## 3. Results

### 3.1. Study Characteristics

Only two studies [29,40] were classified as grey literature, specifically master’s theses or dissertations. Most studies employed a cross-sectional design (n = 13), followed by longitudinal (n = 2), qualitative (n = 2), experimental observation (n = 1), and RCT (n = 1). Sample sizes ranged from 6 to 2281. Three studies included only adolescents and assessed attachment as a general tendency, attachment to parents in general, and attachment to the primary caregiver specifically. Sixteen studies included adults and assessed attachment as a general behavioral tendency to relate to others (n = 16), attachment to the romantic partner (n = 1), attachment to romantic partners and friends (n = 1), attachment to the mother in particular (n = 1), and two studies did not specify to whom the attachment is measured (n = 2).

Only three studies [26,41,42] used a categorical approach to attachment, allowing a clear-cut comparison of emotional eating between insecure and secure samples. Most quantitative studies used a dimensional approach to attachment, assessing insecurity on a continuum. They focused on the relationship between emotional eating and insecurity in both its avoidance and anxiety aspects. They evaluated the attachment styles mostly using the ECR in its different versions [27,28,40,43,44,45,46] or the RQ [41,47] and the RAAS [29], and the attachment state using the State Attachment Anxiety Model (SAAM) [29,47]. Among the qualitative studies, one utilized the RQ [48]. At the same time, the other explored attachment representations within the early family context through interviews [49], emphasizing the participants’ experiential narratives and subjective interpretations of attachment in relation to emotional eating.

Emotional eating was mainly assessed using self-report measures such as The Emotional Eating Scale developed by Arnow, Kenardy, & Agras [28,29,41,43,44,50]; the Three Factor Eating Questionnaire [40,45,46,47,51]; the Dutch Eating Behaviour Questionnaire by Van Strien et al. [26,27,29,42,52], and The Adult Eating Behavior Questionnaire [48]. All these tools focused on negative emotional eating.

Studies were based mainly in the Western industrialized countries: USA (n = 8), Canada (n = 3), Australia (n = 2), UK (n = 2), Greece (n = 1), Ireland (n = 1), and Netherlands (n = 1) and one in the Middle East, Lebanon (n= 1).

All studies assessed both males and females, except for [19,27], which targeted only females. However, none of the studies compared males and females in their analysis of the relationship between attachment and emotional eating.

### 3.2. Attachment Security and Emotional Eating

Studies assessing general attachment security style have found consistent inverse associations between secure attachment and emotional eating. For instance, Maras used the ARQ to measure global attachment both categorically and continuously and found a significant negative correlation between secure attachment and EE (r = −0.223, *p* < 0.001) [42]. Similarly, Zakhour, using a dimensional state measure of attachment (SAAM), reported a significant negative correlation between state secure attachment and EE (r = −0.148, *p* < 0.001) [29]. Fallon used the Relationship Questionnaire (RQ) and generated a continuous score on a general secure attachment style as a category, and found that general secure attachment style was positively associated with emotional eating anxiety scores (EES-Anx) (r = 0.32, *p* < 0.05) [41]. Hierarchical regression analysis revealed that secure attachment style accounted for 9% of the variance in EE-anxiety (F(1, 71) = 8.14, *p* < 0.05, R^2^ = 0.103, Adj. R^2^ = 0.09), indicating a modest but significant predictive role of attachment security style in emotional eating. These findings suggest that individuals who generally perceive themselves as securely attached may be less prone to use food as an emotional regulation strategy.

However, studies focusing on specific attachment relationships, such as attachment to one’s mother in early life or to romantic partners in adulthood, have shown inconsistent results. Beijers et al.’s longitudinal study found non-significant associations between attachment to the mother during toddlerhood (assessed using SSSP and S-AQS) and emotional eating at adolescence, except for a moderate effect when using the home-based S-AQS at age 16 (r = 0.28, *p* < 0.01) [26]. By contrast, Wilkinson et al. found that secure attachment of adults to their romantic partner predicted lower stress-induced eating (β = 0.16, SE = 0.03, *p* < 0.001), indicating the relevance of current close relational bonds in emotional regulation through eating [45].

### 3.3. Attachment Anxiety and Emotional Eating

Studies assessing attachment anxiety as a general bonding style— using dimensional assessment tools such as the ECR-RS [27], RAAS [29], ECR-12 [45,46], SAAM [29], and ECR-16 [28,44]—generally reported a significant association between higher attachment anxiety and greater emotional eating. These findings were consistent across both correlational and predictive models. Taube-Schiff et al. further showed that attachment anxiety is related to specific emotional eating domains, including anger, anxiety, and depression [28]. Experimental evidence from Alexandre (2013) partially supported this link, showing that attachment anxiety predicted increased food intake following social exclusion in one Cyberball study, though subsequent experiments using different manipulations did not replicate this effect [40]. Maras, using the ARQ, also found a significant relationship between general insecure attachment style and emotional eating [42]. However, Leung et al. (2022) was the only study to report no significant association, suggesting some variability across samples or methodological designs [44]. It is also worth noting that the studies by Taube-Schiff et al. [28] and Leung et al. [44] did not mention attachment figures, making it difficult to fully understand the scope of their findings.

Similarly, studies measuring attachment anxiety toward a specific attachment figure consistently report a positive association with emotional eating. Interestingly, all the studies targeted romantic partners as the attachment figure. Wilkinson et al. [23], using the ECR-R targeting romantic relationships, found that attachment anxiety significantly predicted stress-induced eating (β = 0.16, *p* < 0.001) in a mediational model [45]. Similarly, Stapleton and Mackay employed hierarchical multiple regression and found that romantic attachment anxiety significantly predicted emotional eating (B = 0.12, *p* < 0.05), explaining 2% of the variance (adj R^2^ = 0.02, F(1, 224) = 4.79, *p* = 0.03) [47]. Alexandre and Siegel also assessed attachment to romantic partners and friends, not limiting it to one relationship, using the ECR-R and found that attachment anxiety correlated with multiple subscales of the Emotional Eating Scale: EES—Depression (r = 0.289, *p* < 0.01), EES—Anxiety (r = 0.257, *p* < 0.05), and EES—Anger/Frustration (r = 0.194, *p* = 0.068) [43]. These results collectively suggest that attachment anxiety in the context of romantic relationships plays a meaningful role in shaping vulnerability to emotional eating, particularly under conditions of emotional distress.

### 3.4. Attachment Avoidance and Emotional Eating

The relationship between attachment avoidance and emotional eating was generally found to be weak or non-significant across the majority of studies for both general avoidant attachment style and the avoidance of a specific attachment figure. Several studies assessing general attachment styles, such as [27,29,30,40,46], reported no significant correlation between attachment avoidance and emotional eating. Similarly, Alexander and Siegel found no significant associations between attachment avoidance and the Emotional Eating Scale (EES) subscales when measuring attachment to romantic partners and friends [43]. Leung et al., despite a relatively high reported correlation coefficient, found the relationship to be statistically non-significant in their regression model [44]. However, some evidence of a meaningful association emerged when Taube-Schiff et al. addressed specific subscales of emotional eating. He found significant correlations between general attachment avoidance style and all three EES subscales—anger, anxiety, and depression [28]. Finally, only one study, Stapleton and Mackay, using measures of attachment to romantic partners, found that attachment avoidance significantly predicted emotional eating, albeit with a small negative coefficient [47].

Overall, the findings suggest that while attachment avoidance is not consistently related to emotional eating, there may be nuanced associations under specific emotional contexts (e.g., anxiety-driven eating) or when specific relationship domains, such as romantic attachment, are considered. Nevertheless, compared to attachment anxiety, the evidence linking attachment avoidance to emotional eating appears weaker and more inconsistent.

### 3.5. Disorganized Attachment and Emotional Eating

Only one study in this review directly examined the relationship between disorganized attachment and emotional eating. Wilkinson et al., using a 9-item Disorganized Attachment Scale to assess general attachment style, found a small but statistically significant positive correlation between disorganized attachment and emotional eating (r = 0.14, *p* < 0.05) [46]. This finding suggests that individuals with higher levels of disorganized attachment tendencies may be slightly more likely to engage in emotional eating. However, given the limited number of studies addressing this attachment dimension, conclusions should be drawn cautiously.

### 3.6. Mediating Variables

**Figure 2:**
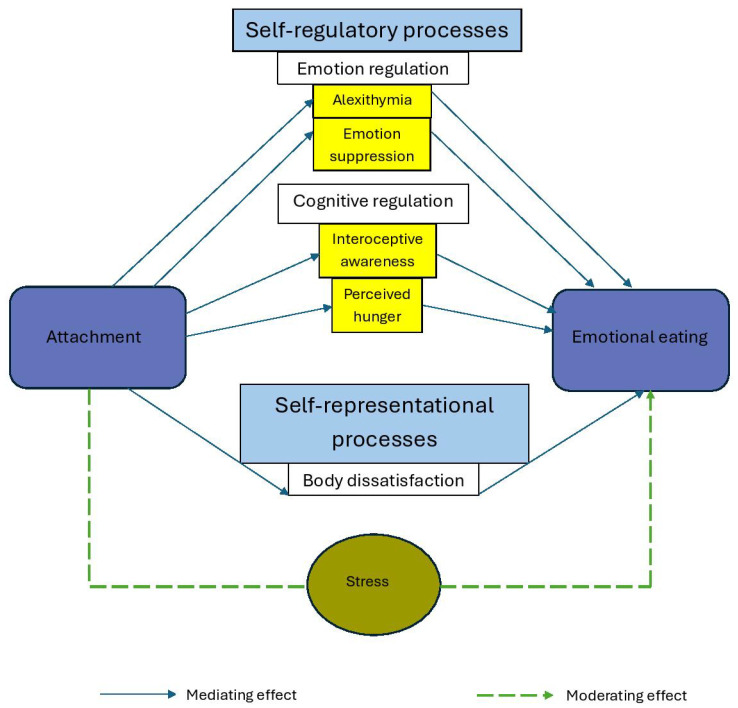
Mediators and moderators linking attachment to emotional eating.

The relationship between attachment and EE is complex and mediated by several psychological processes. Four key mediating variables were identified in the reviewed studies: emotion-regulatory processes, perceived hunger, interoceptive awareness, and body dissatisfaction (see Figure 2).

#### 3.6.1. Emotion-Regulatory Processes

In adolescents, Beijers et al. found that alexithymia and emotion suppression serially mediated the relationship between secure attachment to the mother (measured through home and lab observations) and EE at ages 12 and 16 [26]. The SSSP model showed full mediation (F(5.85) = 7.15, *p* < 0.001, 29.6% variance), while the S-AQS model showed partial mediation (F(5.85) = 9.37, *p* < 0.001, 35.5% variance).

In adults, two studies explored the mediating role of cognitive–emotional regulation. Taube-Schiff et al. found that difficulties in emotion regulation mediated the relationships between attachment avoidance, anxiety, and EE; however, the specific attachment figure was not specified [28]. When the subcategories of emotion regulation were further examined (non-acceptance of emotions, emotional awareness, emotional clarity, access to emotion regulation strategies, impulse control difficulties, and goal-directed behaviors), Wilkinson et al. found that the relationship between general attachment anxiety and stress-induced eating was only mediated by goal-directed behaviors, stressing the cognitive-behavioral dimension of EE [45].

#### 3.6.2. Interoceptive Awareness and Perceived Hunger

Southern found a negative association between interoceptive awareness and insecure attachment to parents, platonic and romantic relationships, but interoceptive awareness did not mediate the relationship between attachment (anxiety or avoidance) and EE [30]. In contrast, perceived hunger—a more subjective interoceptive cue—was investigated as a mediator in three adult studies. Alexander [40] and Alexander & Siegel [43] found that perceived hunger mediated the relationship between general attachment anxiety as well as attachment anxiety to romantic partners and friends and EE subtypes (β = 0.10–0.11), although these effects were not statistically significant (*p* = 0.15–0.29). Stapleton & Mackay, however, found a significant mediation effect (Z = 2.68, *p* = 0.007), suggesting that subjective hunger cues may play a role in mediating the relationship between general attachment and emotional eating in adults [47].

#### 3.6.3. Body Dissatisfaction

Zakhour et al. found that body dissatisfaction mediated 12.53% of the relationship between general attachment security and EE, suggesting that individuals with lower security are more prone to body-related concerns that drive emotional eating [29]. Qualitative data (e.g., Hernandez-Hons & Woolley, [49]) support this mechanism, describing how individuals who are insecurely attached may internalize societal pressures and negative self-images, leading to secretive or emotionally driven eating behaviors.

### 3.7. Moderating Variables

While studies generally support the relationship between attachment insecurity and EE, this connection is not uniform across all contexts. This review showed that stress influences the strength and expression of this relationship.

#### Stress and Coping

Schmitt et al. investigated whether perceived stress moderates the mediating effect of rumination in the association between attachment anxiety and EE [27]. Using the ECR-RS to assess global attachment style, they found that at higher levels of stress, the indirect effect of attachment anxiety on EE through rumination was significant (b = 0.39, SE = 0.17, *p* = 0.03, 95% CI [0.10, 0.79]). However, at lower levels of stress, this indirect effect was not significant (b = 0.21, SE = 0.13, *p* = 0.12, 95% CI [−0.01, 0.50]). This suggests that stress amplifies the impact of attachment anxiety on EE via maladaptive cognitive processes such as rumination.

Qualitative studies showed emotional overeating emerged as an alternative coping strategy when support-seeking was not viable, reflecting how insecurely attached individuals may turn to food as a substitute for emotional solace [48]. This behavior can also be understood in the context of stress-induced eating, where participants reported preoccupation with food, describing it as “calling to them” during times of distress [49]. Moreover, individuals with general insecure attachment histories, including attachment to family, friends, and romantic relationships, as well as those who experienced abuse, often engaged in EE as a way to cope with the pain of failed romantic relationships and the absence of social support [49].

## 4. Discussion

The objective of this review is to explore the relationship between attachment and emotional eating through the lens of different conceptualizations of attachment in adolescents and adults in general populations, and to identify the mediating and moderating factors identified across studies in various countries.

The findings suggest that general secure attachment style is associated with lower levels of emotional eating (EE) in both adolescents and adults, suggesting its potential role as a protective factor against maladaptive eating behaviors. However, this protective effect was less consistent when attachment was assessed toward a specific figure. Conversely, insecure–anxious and disorganized attachment styles were consistently associated with higher EE risk, whether considered as general styles or in relation to specific attachment figures. This aligns with theoretical perspectives proposing that insecure attachment fosters intrapersonal and interpersonal difficulties, prompting individuals to seek comfort through food [53]. However, insecure avoidant attachment showed no significant association with EE, suggesting that avoidant individuals may favor food restraint or avoidance rather than emotional eating.

The significance of this review lies in its exploration of the complex mechanisms explaining the relationship between attachment and EE. Self-regulatory dynamics—emotional regulation difficulties, emotion suppression, alexithymia, and perceived hunger—and self-representational processes such as body satisfaction emerged as important mediators explaining the relationship between attachment and EE, with stress acting as a moderator. These findings align with the emotion regulation perspective, which holds that insecure attachment (anxious, avoidant, or both) often leads to poor distress regulation [54], hindering effective stress coping [53]. Consequently, attachment insecurity undermines emotion regulation, leading to increased reliance on food to manage emotions [55].

However, our findings show that these general linkages are more complex and nuanced. Firstly, the association between attachment and EE is context-dependent. Secure attachment cannot fully protect against EE, as under stressful circumstances, it may not prevent emotional regulation difficulties [56]. Although anxious attachment style was generally positively correlated with EE, in some studies this relationship was observed only when individuals were frustrated [28] or depressed/anxious [29,43]. Furthermore, insecure avoidant attachment showed no significant correlation with EE, except when individuals were anxious. These findings suggest that, regardless of attachment style, in contexts activating stress, frustration, depression, and anxiety, individuals use food to manage emotional distress. Gutierrez-Colina et al. found that emotion regulation difficulties are associated with greater reward-based eating among youth facing adversity [57]. This nuance raises methodological and theoretical questions, as most studies in this review are correlational and may overlook stress as a confounding variable influencing both insecure attachment and EE.

Secondly, specific self-regulatory processes can have different roles in linking attachment to EE. While Braden et al.’s (2025) [58] recent review confirms that lower interoceptive ability—a self-monitoring process allowing for the interpretation of internal cues [59]—is associated with higher emotional eating (EE), particularly in individuals with obesity, our findings suggest that interoceptive awareness did not mediate the relationship between attachment insecurity and EE. Only perceived hunger—a specific internal cue—amplified EE among individuals with high attachment anxiety, lending support to Bruch’s early theory that unresponsive parenting undermines the ability to interpret internal hunger signals [60]. This discrepancy may reflect methodological constraints, as in all the reviewed studies, interoception was assessed broadly across bodily signals supporting homeostasis, many of which are unrelated to eating. However, it can also shed light on the importance of attachment in shaping the relationship with food through early dyadic regulatory processes. In early infancy, regulation primarily occurs at a physiological and sensory level within the caregiver–infant dyad. Through experiences of care, stimulation, and physical closeness, the infant achieves basic physiological satisfaction, feeding and sleep regulation, and a sense of comfort and security. These interactions thus lay the foundation for emotional regulation. The caregiver’s availability and ability to interpret, synchronize, and adapt caregiving behaviors to the infant’s needs act as key regulatory processes, creating a secure, reciprocal interaction that fosters trust in self and others. This review shows that few studies have examined attachment to primary caregivers, with most focusing on general or romantic attachment, leaving the link between attachment and interoceptive awareness in EE largely unexplored.

Thirdly, although evidence highlights the roles of emotional suppression and alexithymia as key mechanisms linking expressed emotion (EE) to obesity [58], in this review, these regulatory processes emerged in only one study of adults. To explain the association between attachment and EE. More studies are needed to explore how attachment in general and insecure avoidance in particular can be a precursor for emotional suppression.

The findings of this review also support the self-representation perspective, which posits that negative self-perceptions linked to insecure attachment drive EE, as individuals use food to cope with feelings of inadequacy [61,62,63]. A recent study highlights guilt as a key factor in dysfunctional eating-related behaviors. Normative guilt is associated with binging and purging, suggesting these behaviors may serve to restore moral integrity as well as control weight. Harm-related guilt predicts interpersonal distrust, indicating that heightened guilt sensitivity may undermine trust due to fears of rejection or causing harm—patterns consistent with insecure attachment. Although emotional eating (EE) is not an eating disorder, it reflects maladaptive affect regulation closely tied to relational processes. Future research should examine guilt as a mediator between attachment insecurity and EE [9]. Moreover, body dissatisfaction mediated 12.53% of the relationship between attachment security and EE, highlighting its significant role. This can be understood because body dissatisfaction reflects the appraisal of a specific dimension of the self, the body. Individuals with a secure attachment style tend to have a positive self-appraisal and low levels of body dissatisfaction [64], a strong sense of self-worth [21], and confidence in their ability to cope with stressors, making them less prone to EE.

Developmental patterns could not be detected due to the disproportionate focus. Only three studies focused on adolescents, while the remaining studies spanned a wide age range (18–83 years), complicating efforts to isolate adolescent-specific effects. Adolescents are particularly susceptible to difficulties in emotion regulation, whereas older adults typically exhibit greater emotional stability [65]. Comparative studies across developmental stages are needed to elucidate how attachment influences EE over time, especially considering the mediating role of self-regulatory processes. Such insights are crucial for designing preventive interventions tailored to the unique needs of different age groups.

Similarly, cultural differences could not be detected due to the disproportionately large number of studies conducted in Western industrialized countries (n  =  18), with only one study from the Middle East. This presents a significant limitation, as both the symbolic and functional roles of food, as well as the nature, expression, and function of attachment, are deeply embedded in cultural contexts. Cultural norms influence how individuals relate to food, including its use in emotional regulation, and shape attachment behaviors and expectations [66,67]. Cultural norms around food may shape emotional eating in distinct ways: in some cultures, food is deeply intertwined with celebrations and social bonding, potentially fostering emotional eating in response to positive emotions, whereas in others, eating is viewed more as a utilitarian act, with emotional eating primarily linked to negative affect [13]. Consequently, findings derived from Western contexts may not be generalizable to non-Western or socioeconomically diverse populations. Future empirical research should examine the differential associations between attachment and both positive and negative emotional eating and situate these relationships within diverse socio-cultural contexts. Such work is essential for clarifying whether cultural norms around food moderate the influence of attachment insecurity on emotional eating patterns. Understanding these dynamics could inform culturally sensitive interventions that address both attachment-related vulnerabilities and emotion-driven eating behaviors.

Finally, interventions on EE, especially when related to obesity, commonly focus on behavioral treatments, particularly cognitive-behavioral and mindfulness-based approaches [58], to target the mechanistic factors explaining EE. Based on our findings, interventions targeting emotional eating (EE) may be most effective when they also incorporate attachment theory within treatment and prevention frameworks. This integrated approach targets the underlying relational and regulatory mechanisms that contribute to EE. Therapeutic programs that aim to foster secure attachment and provide corrective relational experiences—such as emotionally focused therapy (EFT) and attachment-based family therapy—have demonstrated effectiveness in improving emotional regulation and reducing maladaptive coping strategies [68,69]. Preventive interventions during early caregiving are equally important. Programs like Circle of Security and Video-feedback Intervention to Promote Positive Parenting (VIPP) support caregivers in establishing sensitive, responsive interactions that promote secure attachment and self-regulation in children [70,71]. These interventions encourage practices such as attuned feeding and synchronized routines, which lay the foundation for emotional regulation and positive self-representations—protective factors against future emotional eating and obesity risk [58]. Future research should assess the effectiveness of these interventions and prevention programs in reducing EE, specifically in the context of obesity.

## 5. Limitations

At the conceptual level, this review identifies four primary limitations in the current literature exploring the relationship between attachment and emotional eating. First, the majority of studies conceptualize attachment predominantly as an individual’s self-perceived style of relating to others—either in general or with a specific partner—rather than as a representation of early relational experiences with primary caregivers. Among the 19 studies reviewed, only one examined attachment as a reflection of early caregiver-child dynamics employing a qualitative approach. This conceptualization significantly constrains our understanding of how attachment influences emotional eating. Given that regulating food intake is a core function within the caregiver-child relationship, investigating the link between early attachment representations and later emotional eating behaviors could provide critical insights into the developmental origins of maladaptive eating patterns. Studies anchored in attachment theory provide evidence for the association between internal working models of self and world (IWMs) and dysregulated self-control, as well as the cognitive–emotional interactions that underpin eating pathology [72,73].

Second, most studies assess attachment as a general style, overlooking the possibility that the same person can have multiple attachment scripts, each differently activated depending on the circumstances [24]. Moreover, when studies do consider attachment to specific figures, they overwhelmingly focus on romantic partners, thereby neglecting the potential influence of other significant relationships such as those with parents, siblings, or peers. This is a notable oversight, as early caregivers and other close relational figures play a central role in shaping emotional regulation and coping strategies, including those related to food intake. Expanding the scope of research to include these relationships could enrich our understanding of the multifaceted ways in which attachment dynamics contribute to emotional eating. Attachment may then be context-driven, and studying how different contexts activate cognitive-behavioral patterns could reveal a more nuanced link between attachment and EE. Our study highlights a critical gap in the conceptualization of attachment as a foundational factor in emotional eating (EE). We thus concur with Braden et al.’s (2025) observation that the conceptual rigor of psychosocial constructs remains insufficient for fully explaining emotional eating (EE) in the context of obesity [58]. Addressing this gap is essential for advancing theoretical clarity and empirical precision in future research.

Third, most studies inadequately accounted for age as a proxy for pubertal development and failed to test for sex differences in outcomes. On one hand, this position overlooks the centrality of reproductive physiology in explaining the variability in emotional eating between males and females, or within one sex at different stages of reproductive function. New studies emphasize the importance of considering the hypothalamic–pituitary–gonadal (HPG) axis in defining sex-specific changes in appetite and eating behavior, particularly during puberty [34]. On the other hand, this position also overlooks the possible variation of attachment between sexes. Pierrehumbert et al. (2009) found gender differences in attachment; however, the magnitude of these differences varies across countries [74]. Methodological and statistical designs become essential to examine the effect of sex on the relationship between attachment and EE, especially across different cultural contexts and reproductive stages. To achieve a more comprehensive and globally relevant understanding of the attachment–emotional eating connection, further research should encompass a broader range of cultural and socioeconomic contexts.

Fourth, the health consequences of emotional eating vary depending on the valence of the emotion that triggers overeating. A recent meta-analysis by Nolan et al. (2025) suggests that negative emotional eating (NEE) is associated with a higher body mass index (BMI) [13]. In contrast, positive emotional eating (PEE) tends to correlate with a lower BMI. These findings suggest that NEE may be more strongly linked to difficulties in emotion regulation and the consumption of energy-dense foods, which could explain its association with elevated BMI. To deepen our understanding of these dynamics, future research should examine the differential relationships between attachment and both positive and negative forms of emotional eating, thereby clarifying the complexity of underlying mechanisms and informing targeted interventions.

From a methodological standpoint, most of the reviewed studies adopt cross-sectional designs, which limit the ability to infer causal or developmental pathways linking attachment and emotional eating. Longitudinal research is needed to elucidate how early attachment experiences with caregivers influence the emergence and persistence of emotional eating behaviors over time. Such studies would offer a more comprehensive picture of the relational roots of emotional eating and inform early intervention strategies.

Moreover, our review underscores the considerable heterogeneity in the instruments used to assess both emotional eating and attachment. Commonly employed measures of emotional eating—such as the Three-Factor Eating Questionnaire (TFEQ), Dutch Eating Behavior Questionnaire (DEBQ), and Emotional Eating Scale (EES)—differ markedly in their conceptualization of the EE construct, the range of emotions they capture, and their scoring systems. Similarly, attachment assessments vary in methodological approach (dimensional vs. categorical), temporal focus (past experiences with caregivers vs. current relationships vs. generalized attachment style), and the level of specificity (a global bonding style vs. attachment to a specific significant other). This variability complicates cross-study comparisons and highlights the need for greater conceptual and methodological consistency. In addition, while the majority of studies provided clear definitions of their conceptual frameworks, research objectives, and methodological designs, sample-related biases are prevalent across the literature. Notably, most studies did not justify their sampling frame or selection procedures. This lack of transparency may limit the representativeness of the study populations, thereby constraining the generalizability of the findings and the validity of the researchers’ inferences.

Finally, none of the studies address the bidirectional relationship between emotional eating (EE) and attachment, which is crucial for advancing theoretical and clinical understanding. While insecure attachment and maladaptive internal working models (IWMs) can predispose individuals to EE, persistent engagement in EE may, in turn, reinforce or reshape attachment representations through self-representational mechanisms, creating a self-perpetuating cycle. This dynamic interplay underscores the need for future research to adopt longitudinal and transactional designs that capture these reciprocal effects. Such an approach could inform interventions that simultaneously target attachment-related vulnerabilities and maladaptive coping strategies, ultimately promoting more sustainable outcomes in the prevention of eating pathology.

## 6. Conclusions

This review provides a comprehensive analysis of studies from 1990 to 2025 and captures recent advancements in understanding the relationship between attachment and emotional eating. Incorporating both quantitative and qualitative methods, it offers a multidimensional perspective. However, with only 2 qualitative studies among the 19 (Table S4), quantitative research predominates, underscoring the need for further qualitative exploration. Focusing on the general population enhances applicability to non-clinical settings, thereby contributing to the prevention of EE in non-clinical contexts. Additionally, including grey literature reduces publication bias, though peer-reviewed articles remain overrepresented.

Although inconsistent EE definitions and varying methods across studies affected the comparability between the studies, this review highlights the significant link between insecure anxious attachment style and EE, emphasizing the importance of early prevention in adolescents and adults. Key mediators include emotion regulation, perceived hunger, and body dissatisfaction, while stress serves as a moderator that further elucidates attachment’s role in emotional eating. The review identifies critical gaps, including the need for a more comprehensive exploration of non-Western, low-income populations, gender-specific issues, and factors related to pubertal development. Future research should investigate additional psychological and environmental mediators and address methodological issues, particularly confounding variables that may activate specific attachment patterns and overlook the complexity of the attachment–EE relationship. Future research should also examine how representations of attachment to early caregivers shape relationships with emotional eating, building on prior evidence that internal working models (IWMs) are strongly linked to disordered eating [72,73]. It is also worth exploring representations of attachment to early caregivers in their relationships with EE. This work could contribute to Sustainable Development Goal (SDG) 3, which aims to ensure healthy lives and promote well-being for all at all ages. By giving insights into how attachment relationships influence eating behaviors and overall health, this study supports efforts to address both mental and physical health challenges in the general population. Interventions targeting emotional eating (EE) should combine strategies that enhance self-regulatory processes with attachment-informed approaches addressing underlying relational mechanisms. Preventive and therapeutic programs that foster secure attachment may be promising for reducing reliance on food as a coping strategy and promoting long-term physical and emotional health.

## Data Availability

The data analyzed in this review consist of previously published studies that are publicly accessible. All relevant data supporting the findings of this review can be found within the cited studies listed in the references and is available upon request.

## Supplementary Materials

The following supporting information can be downloaded at: https://www.mdpi.com/article/10.3390/healthcare13233170/s1. Table S1: Risk of bias assessment for included studies; Table S2: Table for quantitative studies on adolescents; Table S3: Table for quantitative studies on adults; Table S4:Table for qualitative studies.

## Abbreviations

The following abbreviations are used in this manuscript:

ARQ	Adolescent Relationship Questionnaire
ECR	Experiences in Close Relationships scale
ECR-R	Experiences in Close Relationships Scale-Revised
EE	Emotional Eating
EES	Emotional Eating Scale
EES-AF	Angry/Frustrated Eating
EES-Anx	Anxious Eating
EES-DEP	Depressive Eating
EO	Emotional Overeating
EU	Emotional Undereating
RAAS	The Revised Adult Attachment Scale
RQ	Relationship Questionnaire
SAAM	State Attachment Anxiety Model
S-AQS	The Shortened Version of the Attachment Q-Set
SSSP	The Shortened Strange Situation Procedure

## References

[1] Wansink B., Payne C. (2007). Mood self-verification explains the selection and intake frequency of comfort foods. Adv. Consum. Res..

[2] Chew H.S.J., Soong R.Y., Ang W.H.D., Ngooi J.W., Park J., Yong J.Q.Y.O., Goh Y.S.S. (2025). The global prevalence of emotional eating in overweight and obese populations: A systematic review and meta-analysis. Br. J. Psychol..

[3] van Strien T., Engels R.C.M.E., van Leeuwe J., Snoeck H.M. (2005). The Stice model of overeating: Tests in clinical and non-clinical samples. Appetite.

[4] Sultson H., Kukk K., Akkermann K. (2017). Positive and negative emotional eating have different associations with overeating and binge eating: Construction and validation of the positive-negative emotional eating scale. Appetite.

[5] Grant P.G., Boersma H. (2005). Making sense of being fat: A hermeneutic analysis of adults’ explanations for obesity. Couns. Psychother. Res..

[6] Alagha M., Al-Alam F., Saroufine K., Elias L., Klaimi M., Nabbout G., Harb F., Azar S., Nahas N., Ghadieh H.E. (2025). Binge eating disorder and metabolic syndrome: Shared mechanisms and clinical implications. Healthcare.

[7] Evers C., Dingemans A., Junghans A.F., Boevé A. (2018). Feeling bad or feeling good, does emotion affect your consumption of food? A meta-analysis of the experimental evidence. Neurosci. Biobehav. Rev..

[8] Herle M., Fildes A., Steinsbekk S., Rijsdijk F., Llewellyn C.H. (2017). Emotional over- and under-eating in early childhood are learned not inherited. Sci. Rep..

[9] Raffone F., Atripaldi D., Barone E., Marone L., Carfagno M., Mancini F., Saliani A.M., Martiadis V. (2025). Exploring the role of guilt in eating disorders: A pilot study. Clin. Pract..

[10] Dakanalis A., Mentzelou M., Papadopoulou S.K., Papandreou D., Spanoudaki M., Vasios G.K., Pavlidou E., Mantzorou M., Giaginis C. (2023). The association of emotional eating with overweight/obesity, depression, anxiety/stress, and dietary patterns: A review of the current clinical evidence. Nutrients.

[11] Stroebe W., Papies E.K., Aarts H. (2008). From homeostatic to hedonic theories of eating: Self-regulatory failure in food-rich environments. Appl. Psychol..

[12] Godet A., Fortier A., Bannier E., Coquery N., Val-Laillet D. (2022). Interactions between emotions and eating behaviors: Main issues, neuroimaging contributions, and innovative preventive or corrective strategies. Rev. Endocr. Metab. Disord..

[13] Nolan L.J., Barnhart W.R., Diorio G., Gallo V., Geliebter A. (2025). A systematic review and meta-analysis of cross-sectional questionnaire studies of the relationship between negative and positive emotional eating and body mass index: Valence matters. Appetite.

[14] Bowlby J. (1982). Attachment and Loss: Attachment.

[15] Ainsworth M.D., Blehar M.C., Waters E., Wall S. (1978). Patterns of Attachment: Assessed in the Strange Situation and at Home.

[16] Main M., Solomon J., Greenberg M.T., Cicchetti D., Cummings E.M. (1990). Procedures for identifying infants as disorganized/disoriented during the Ainsworth Strange Situation. Attachment in the Preschool Years: Theory, Research, and Intervention.

[17] Vaughn B.E., Waters E. (1990). Attachment Behavior at Home and in the Laboratory: Q-Sort Observations and Strange Situation Classifications of One-Year-Olds. Child Dev..

[18] Main M., Kaplan N., Cassidy J. (1985). Security in infancy, childhood, and adulthood: A move to the level of representation. Monogr. Soc. Res. Child Dev..

[19] Pierrehumbert B., Karmaniola A., Sieye A., Meister C., Miljkovitch R., Halfon O. (1996). Les modèles de relations: Développement d’un autoquestionnaire d’attachement pour adultes. Psychiatr. Enfant.

[20] Varley D., Sherwell C.S., Kirby J.N. (2024). Attachment and propensity for reporting compassionate opportunities and behavior in everyday life. Front. Psychol..

[21] Bartholomew K., Horowitz L.M. (1991). Attachment styles among young adults: A test of a four-category model. J. Pers. Soc. Psychol..

[22] Collins N.L., Read S.J. (1990). Adult attachment, working models, and relationship quality in dating couples. J. Pers. Soc. Psychol..

[23] Fraley R.C., Waller N.G., Brennan K.A. (2000). An item response theory analysis of self-report measures of adult attachment. J. Pers. Soc. Psychol..

[24] Waters H.S., Waters E. (2006). The attachment working model’s concept: Among other things, we build script-like representations of secure base experiences. Attach. Hum. Dev..

[25] Gillath O., Hart J., Noftle E.E., Stockdale G.D. (2009). Development and validation of a state adult attachment measure. J. Res. Pers..

[26] Beijers R., Miragall M., van den Berg Y., Konttinen H., van Strien T. (2021). Parent–infant attachment insecurity and emotional eating in adolescence: Mediation through emotion suppression and alexithymia. Nutrients.

[27] Schmitt A.P., Hart E., Chow C.M. (2020). Attachment, rumination, and disordered eating among adolescent girls: The moderating role of stress. Eat. Weight Disord..

[28] Taube-Schiff M., Van Exan J., Tanaka R., Wnuk S., Hawa R., Sockalingam S. (2015). Attachment style and emotional eating in bariatric surgery candidates: The mediating role of difficulties in emotion regulation. Eat. Behav..

[29] Zakhour M., Haddad C., Salameh P., Sacre H., Hallit R., Akel M., Kheir N., Hallit S., Obeid S. Association between adult attachment styles and disordered eating among a sample of Lebanese adults. Res. Sq..

[30] Southern M. (2020). Understanding the Association Between Attachment, Interoceptive Awareness, and Unhealthy Eating Behaviours. Master’s Thesis.

[31] Cortés-García L., Rodríguez-Cano R., von Soest T. (2022). Prospective associations between loneliness and disordered eating from early adolescence to adulthood. Int. J. Eat Disord..

[32] Faber A., Dubé L., Knäuper B. (2018). Attachment and eating: A meta-analytic review of the relevance of attachment for unhealthy and healthy eating behaviors in the general population. Appetite.

[33] United Nations (2015). Transforming Our World: The 2030 Agenda for Sustainable Development (A/RES/70/1).

[34] Geary N., Asarian L., Leeners B. (2025). Best practices for including sex as a variable in appetite research. Appetite.

[35] Smink F.R., van Hoeken D., Hoek H.W. (2012). Epidemiology of eating disorders: Incidence, prevalence and mortality rates. Curr. Psychiatry Rep..

[36] Jansen A., van Strien T. (2005). The influence of body dissatisfaction on emotional eating in adolescents. Eat. Behav..

[37] Evers C., Stok F.M., de Ridder D.T.D. (2010). Emotional eating: Food as a means to regulate mood. Handbook of Behaviorism.

[38] Deng B. (2015). Why Is English the Language of Science? Slate Magazine. https://slate.com/technology/2015/01/english-is-the-language-of-science-u-s-dominance-means-other-scientists-must-learn-foreign-language.html.

[39] Ouzzani M., Hammady H., Fedorowicz Z., Elmagarmid A. (2016). Rayyan—A web and mobile app for systematic reviews. Syst. Rev..

[40] Alexander K.E. (2013). Attachment Insecurity and Emotional Eating. Doctoral Dissertation.

[41] Fallon C. (2012). A Study Investigating the Relationship Between Adult Attachment, Eating Behaviour, Weight, and Emotional Expression in Childhood. Master’s Thesis.

[42] Maras D. (2013). Attachment Style and Obesity: Examination of Eating Behaviours as Mediating Mechanisms in a Community Sample of Ontario Youth.

[43] Alexander K.E., Siegel H.I. (2013). Perceived hunger mediates the relationship between attachment anxiety and emotional eating. Eat. Behav..

[44] Leung S.E., Wnuk S., Cassin S., Jackson T., Hawa R., Sockalingam S. (2019). Prospective study of attachment as a predictor of binge eating, emotional eating and weight loss two years after bariatric surgery. Nutrients.

[45] Wilkinson L.L., Rowe A.C., Robinson E., Hardman C.A. (2018). Explaining the relationship between attachment anxiety, eating behaviour and BMI. Appetite.

[46] Wilkinson L.L., Rowe A.C., Millings A. (2019). Disorganized attachment predicts body mass index via uncontrolled eating. Int. J. Obes..

[47] Stapleton P., Mackay E. (2014). Psychological determinants of emotional eating: The role of attachment, psychopathological symptom distress, love attitudes and perceived hunger. Curr. Res. Psychol..

[48] Mamo H.I., Louka P. (2022). The experience of emotional eating in individuals with insecure attachment style: An interpretative phenomenological analysis (IPA) approach. Dialogues Clin. Neurosci. Ment. Health.

[49] Hernandez-Hons C., Woolley S. (2011). Women’s experiences with emotional eating and related attachment and sociocultural processes. J. Marital Fam. Ther..

[50] Arnow B., Kenardy J., Agras W.S. (1995). The emotional eating scale: The development of a measure to assess coping with negative affect by eating. Int. J. Eat Disord..

[51] Stunkard A.J., Messick S. (1985). The three-factor eating questionnaire to measure dietary restraint, disinhibition and hunger. J. Psychosom. Res..

[52] van Strien T., Frijters J.E.R., Bergers G.P.A., Defares P.B. (1986). The Dutch Eating Behavior Questionnaire (DEBQ) for assessment of restrained, emotional, and external eating behavior. Int. J. Eat Disord..

[53] Mikulincer M., Shaver P.R. (2007). An attachment perspective on interpersonal regulation. Attachment in Adulthood: Structure, Dynamics, and Change.

[54] Bowlby J. (1979). The Bowlby-Ainsworth attachment theory. Behav. Brain Sci..

[55] Nolen-Hoeksema S. (2012). Emotion regulation and psychopathology: The role of gender. Annu. Rev. Clin. Psychol..

[56] Domic-Siede M., Guzmán-González M., Sánchez-Corzo A., Álvarez X., Araya V., Espinoza C., Zenis K., Marín-Medina J. (2024). Emotion regulation unveiled through the categorical lens of attachment. BMC Psychol..

[57] Gutierrez-Colina A.M. (2025). Self-regulation and behavioural risk factors for obesity in youth facing adversity: Emotion regulation difficulties are related to greater reward-based eating and sleep disturbances in youth facing adversity. Obes. Med..

[58] Braden A., Ahlich E., Koball A.M. (2025). Emotional eating and obesity: An update and new insights. Curr. Obes. Rep..

[59] Nelson T.O., Narens L., Bower G. (1990). Metamemory: A theoretical framework and new findings. The Psychology of Learning and Motivation.

[60] Bruch H. (1964). Psychological aspects of overeating and obesity. Psychosomatics.

[61] Bento C., Pereira A.T., Maia B., Marques M., Soares M.J., Bos S., Macedo A. (2010). Perfectionism and eating behaviour in Portuguese adolescents. Eur. Eat. Disord. Rev..

[62] Ferreira C., Pinto-Gouveia J., Duarte C. (2014). Self-criticism, perfectionism and eating disorders: The effect of depression and body dissatisfaction. Int. J. Psychol. Psychol. Ther..

[63] Shafran R., Mansell W. (2001). Perfectionism and psychopathology: A review of research and treatment. Clin. Psychol. Rev..

[64] Homan K.J., Wild S., Dillon K.R., Shimrock R. (2017). Don’t bring me down. J. Soc. Pers. Relatsh..

[65] Zimmermann P., Iwanski A. (2014). Emotion regulation from early adolescence to emerging adulthood and middle adulthood: Age differences, gender differences, and emotion-specific developmental variations. Int. J. Behav. Dev..

[66] Keller H. (2018). Universality claim of attachment theory: Children’s socioemotional development across cultures. Proc. Natl. Acad. Sci. USA.

[67] Nahas N. (2020). Liens d’attachement: Une autre perspective pour une autre culture. Étude exploratoire sur des enfants libanais. Enfance.

[68] Crane M., Patterson E. (2024). Emotion-Focused Therapy for Eating Disorders.

[69] Dolhanty J., Lafrance A. (2019). Emotion-focused family therapy for eating disorders. Clinical Handbook of Emotion-Focused Therapy.

[70] Powell B., Cooper G., Hoffman K., Marvin B. (2014). The Circle of Security Intervention: Enhancing Attachment in Early Parent–Child Relationships.

[71] Topham G.L. (2019). The Circle of Security Intervention: Building Early Attachment Security.

[72] Barca L., Pezzulo G. (2020). Keep your interoceptive streams under control: An active inference perspective on Anorexia Nervosa. Cogn. Affect. Behav. Neurosci..

[73] Pezzulo G., Barca L., Friston K. (2015). Active inference and cognitive-emotional interactions in the brain. Behav. Brain Sci..

[74] Pierrehumbert B., Santelices M.P., Ibáñez M., Alberdi M., Ongari B., Roskam I., Stievenart M., Spencer R., Rodríguez A.F., Borghini A. (2009). Gender and attachment representations in the preschool years: Comparisons between five countries. J. Cross-Cult. Psychol..

