# Supplementary material for: Attachment and Emotional Eating: A Scoping Review Uncovering Relational Roots to Inform Preventive Healthcare
Source: Healthcare (Basel). 2025 Dec 4;13(23):3170. doi: 10.3390/healthcare13233170 (PMC12692234; doi:10.3390/healthcare13233170)
Supplement: Supplementary file 1 [file healthcare-13-03170-s001.zip › healthcare-3997960-supplementary.pdf]

Table S1. Risk of Bias Assessment for Included Studies

Study (Author, Year)	Study Design	Risk of Bias Tool Used	Final rating	Risk of Bias Rating
Beijers et al. 2021 [26]	Longitudinal	Newcastle-Ottawa Quality Assessment Form for Cohort Studies	7/9	Low
Maras, 2013 [42]	Cross-sectional	AXIS	16/20	Low
Schmitt et al., 2020 [27]	Cross-sectional	AXIS	13/20	Moderate
Alexander, 2013 (study 1) [40]	Cross-sectional	AXIS	16/20	Low
Alexander, 2013 (study 2) [40]	Cross-sectional	AXIS	13/20	Moderate
Alexander, 2013 (study 3) [40]	Cross-sectional	AXIS	13/20	Moderate
Alexander, 2013 (study 4) [40]	RCT	RoB 2	No numerical rating	Some concerns
Alexander & Siegel, 2013 [43]	Cross-sectional	AXIS	12/20	Moderate
Fallon, 2012 [41]	Cross-sectional	AXIS	13/20	Moderate
Leung et al., 2019 [44]	Longitudinal	Newcastle-Ottawa Quality Assessment Form for Cohort Studies	6/9	Moderate
Stapleton & Mackay, 2014 [47]	Cross-sectional	AXIS	13/20	Moderate
Southern, 2020 [30]	Cross-sectional	AXIS	16/20	Low
Taube-Schiff et al., 2015 [28]	Cross-sectional	AXIS	15/20	Low
Wilkinson et al., 2018 (study 1) [45]	Cross-sectional	AXIS	16/20	Low
Wilkinson et al., 2018 (study 2) [45]	Cross-sectional	AXIS	16/20	Low
Wilkinson et al., 2019 [46]	Cross-sectional	AXIS	17/20	Low
Zakhour et al., 2021 [29]	Cross-sectional	AXIS	16/20	Low
Mamo & Louka, 2022 [48]	Qualitative	CASP	8/9	Low
Hernandez-Hons & Woolley, 2011 [49]	Qualitative	CASP	9/9	Low

Note: AXIS= Appraisal tool for Cross-Sectional Studies, CASP= Critical Appraisal Skills Programme checklist, RCT= Randomized Controlled Trials, RoB 2= Risk of Bias version 2.

Table S2. Table for Quantitative Studies on Adolescents

Authors (year, country)	Study design / Sample	Measures	Results
Beijers et al. (2021), Netherlands [26]	LO; 91 adolescents. Wave 1 at M=12.4 years; Wave 2 at M=16.3 years. Primary caregivers aged 24–47 (M=32.9, SD=4.4).	**EE:** DEBQ<br><br>**Attachment:** Categorical continuous attachment (SSSP, 15–28 months); Home observation (S-AQS, 15–28 months; attachment to mothers).<br><br>**Mediators:** Suppression of emotions, Alexithymia.	Association At 12 SA (SSSP) and EE, r=0.1 (not significant) SA (S-AQS) and EE, r=0.07 (not significant)<br><br>Association At 16 SA (SSSP) and EE, r=0.14 (not significant) SA (S-AQS) and EE, r=0.28, p<0.01<br><br>Serial mediation At 12 years old: SA (S-AQS): F (5.98) =6.27, p<0.001; 24.25% of variance in EE.<br><br>Serial mediation: At 16 years old: SA (SSSP) and EE: F (5.85) =7.15, p<0.001; 29.6% of variance in EE.<br><br>Serial mediation: At 16 years old: SA (S-AQS) and EE: F (5.85) = 9.37, p<0.001); 35.5% of variance in EE.

Maras, 2013, Canada [42]	CS; *N* = 2,281 adolescents, aged 11.08–20.75 years (*M* = 14.08, *SD* = 1.57); 966 males, 1,315 females.	**EE:** DEBQ.<br><br>**Attachment:** Categorical, dichotomous, and continuous attachment using ARQ; (global attachment style).<br><br>**Controls:** Age and SES.	Correlation: SA and EE (r= -0.223), p< 0.001 in the normal sample<br><br>IA and EE (r= 0.179), p < 0.001 in the normal sample
Schmitt et al. (2020), USA [27]	CS; *N* = 100 adolescents (*M* = 14.35, *SD* = 2.29); all females.	**EE:** DEBQ.<br><br>**Attachment:** Dimensional attachment using ECR-RS; (global attachment style).<br><br>**Mediators/Moderators:** Rumination (mediator), Stress (moderator).	Correlation: A-Anx and EE: (r= 0.42), p<0.05<br><br>Correlation: A-Avoid and EE: r=0.06 (not significant)<br><br>Mediation: A-Anx and rumination on EE (B= 0.07, SE= 0.03, p= 0.02, LL= 0.02, UL= 0.16).<br><br>Mediation: A-Avoid and rumination on EE (B= 0.02, SE= 0.02, p= 0.37, LL= -0.02, UL= 0.07).<br><br>Moderated mediation: Stress, higher levels of A-Anx through rumination on EE (bindirect = 0.39, SE = 0.17, p

			= 0.03, CI 95 = 0.10 to 0.79). Stress, lower levels of A-Anx through rumination on EE:(bindirect = 0.21, SE = 0.13, p = 0.12, CI95 = − 0.01 to 0.50)

Table S3 Table for Quantitative Studies on Adults

Authors (year, country)	Study design / Sample	Measures	Results
Alexander, 2013, USA [40]	Study 1: CS; N = 97; Age 18–50, M = 20.69 (SD = 4.94); 37 males, 60 females	**EE**: TFEQ; **Attachment**: Dimensional attachment using ECR-R (global attachment); **Mediator**: Perceived hunger	Correlation: A-Anx and EES-Anx ($r = .257$, $p < .05$) Correlation: A-Anx and EES-Dep ($r = .289$, $p < .01$) Correlation: EE and A-Avoid (not significant) Correlation: Disorganized attachment and EE ($r=.14$ with $p<.05$) Mediation: Perceived hunger mediated A-Anx and EES-Dep ($\beta=0.11, p=0.15$, $R^2=0.54$). Mediation:

			Perceived hunger mediated between A-Anx and EES- Anx (β=0.10, p=0.29, R 2=0.37).
	Study 2: CS; N = 72; Age 18–66, M = 21.7 (SD = 7.95); 28 males, 44 females	**EE**: TFEQ;<br><br>**Attachment**: Dimensional attachment using ECR-R and SAAM (global attachment)	Food consumption was not related to A-Avoid<br><br>Ostracism did not predict EE or interact with A-Anx
	Study 3: Experimental condition of ostracism; N = 103; Age 17–52, M = 21 (SD = 4.79); 38 males, 65 females	**EE**: TFEQ; Observational measure of eating behavior;<br><br>**Attachment**: Dimensional attachment using ECR-R and SAAM (global attachment)	Regression analysis:<br>A-Anx did not predict with increased food consumption after ostracism F (2,102) = 0.20, p=.83, R2=.004 → Not significant
	Study 4: RCT; N = 206; Age 18–53, M = 21; 64 males, 142 females; Priming attachment security through journal group (attachment, positive, neutral) (independent); Condition (ostracized, included) (moderator)	**EE**: TFEQ;<br><br>**Attachment**: Dimensional attachment using ECR-R and SAAM (global attachment)	Moderated multiple regression:<br>No direct effects of the journal interventions or global A-Anx on amount of food eaten
Alexander & Siegel, 2013, USA [43]	CS; N = 97; Age 16–35, M = 20.69 (SD = 4.94); 37 males, 60 females	**EE**: EES;<br><br>**Attachment**: Dimensional attachment using ECR-R (attachment to romantic	Correlation:<br>A-Anx and EES-Dep (r = .289, p< .01)<br><br>Correlation:<br>A-Anx and EES-Anx (r = .257, p< .05)

		partners and friends);<br><br>**Mediator**: Perceived hunger	Correlation: A-Anx and EES-AF (r = .194, p = .068)<br><br>Correlation: A-Avoid and EES-Dep (r=0.156), EES-Anx (r=.08), EES-AF (r=.059) (not significant)<br><br>Mediation: Perceived hunger mediated between A-Anx and EES-Dep (β = 0.11, p = 0.15, R^2 = 0.54)<br><br>Mediation: Perceived hunger mediated between A-Anx and EES-Anx (β = 0.10, p = 0.29, R^2 = 0.37)
Fallon, 2012, Ireland [41]	CS; N = 76; Age 19–59, M = 30; 62 females, 14 males	**EE**: EES;<br><br>**Attachment**: Categorical attachment continuous using RQ (global attachment)	Correlation: Attachment style and EES-ANX (r = .32, n = 73, p < .05).<br><br>Hierarchical regression: Attachment style accounted for 9% of variance in the prediction of EES-Anx (F(1,71)= 8.14, p < .05, R^2 = .103, AdjR^2 = .09).

Leung et al., 2019, Canada [44]	LO; N = 108; Age 18–65; 84.3% female, 15.7% male	**EE**: EES;<br><br>**Attachment**: Dimensional attachment using ECR-16 (not specified)	Multivariate linear regression: A-Avoid and EE: r=0.407, p=0.686 (not significant)<br><br>Multivariate linear regression: A-Anx and EE: r=1.347, p= 0.179 (not significant)
Stapleton & Mackay, 2014, Australia [47]	CS; N = 226 currently in a romantic relationship; Age 18+; 197 females, 29 males	**EE**: TFEQ;<br><br>**Attachment**: Dimensional attachment using RQ and SAAM (global attachment);<br><br>**Mediator**: Perceived hunger	Hierarchical Multiple linear regression: A-Anx predicted EE (B= 0.12, p< 0.05).<br><br>Hierarchical Multiple linear regression: A-Avoid predicted EE (B= -0.09).<br><br>Hierarchical Multiple linear regression: A-Anx scores 2% of the variability in EE scores (adj R2= 0.02, F(1.224)= 4.79; p= 0.03).<br><br>Hierarchical Mutiple linear regression: Higher A-Anx scores predicted higher EE scores.

			(B = 0.02, SE B = 0.01, β = 0.15, p = 0.030).<br><br>Mediation: Perceived hunger mediated the relationship between A-Anx and EE. Sobel test: Z = 2.68, p = 0.007.
Southern, 2020, Australia [30]	CS; N = 216; Age 18–83, M = 30.02 (SD = 14.75); 73.6% females, 26.4% males	**EE**: DEBQ;<br><br>**Attachment**: Dimensional attachment using RAAS (attachment to parents, platonic, and romantic relationships);<br><br>**Mediator**: Interoceptive awareness	Correlation: A-Anx and EE: (r= 0.316, p<0.001).<br><br>Correlation: A-Avoid and EE: (r=0.131, p<0.06).<br><br>Mediation: Interoceptive awareness did not mediate between A-Anx and A-Avoid on EE
Taube-Schiff et al., 2015, Canada [28]	CS; N = 1393; Age 18+, M = 44.72; 288 males, 1095 females	**EE**: EES;<br><br>**Attachment**: Dimensional attachment using ECR-16 (Brennan, Clark, & Shaver, 1998) (not specified);<br><br>**Mediator**: Difficulties in emotion regulation	Path analysis within a structural equation modeling (SEM) A-Anx and EES-AF (B=.08, SE=.03, p<0.01). Path analysis within a structural equation modeling (SEM)

			A-Avoid and EES-Anx (B=.05, SE=.04, p<0.01).<br><br>Mediation: DER mediator between A-Avoid, A-Anx and EES-AF, EES-Anx, and EES-Dep (ab ranging from .02 to .05, all p-values b .001)
Wilkinson et al., 2018, UK & USA [45]	Study 1: CS; N = 665; Age 18+, M = 28.8 (SD = 13.5); 144 males, 521 females	**Attachment**: Dimensional, continuous using ECR (global attachment) (Lafontaine, 2016, 12 items)	Direct effect in a mediational model (ttesting if EE is a mediator between attachment and BMI) A-Anx and EE (b=5.08, SE=0.78, p<.001)
	Study 2: CS; N = 548; Age 18+, M = 36 (SD = 11.9); 237 males, 311 females	**EE**: TFEQ;<br><br>**Attachment**: Dimensional, continuous using ECR-R (36 items; Fraley, Waller, & Brennan, 2000, attachment to romantic partners);<br><br>**Mediator**: Difficulties in emotion regulation	Mediation: Non-acceptance, awareness, clarity, strategies, and impulse (all dimensions of dysregulation) did not mediate between A-Anx and stress-induced eating (p-values > .05), Mediation: Goal-directed behaviors mediated between A-Anx and stress-induced eating (β=0.03, p=.004).

			Mediation: SA predicted stress-induced eating (b=0.16, SE= 0.03, p < .001) Direct effect in a mediational model: A-Anx and stress-induced eating (β=0.16, p<.001)
Wilkinson et al., 2019, UK [46]	CS; N = 537; M = 25.5 (SD = 9.9); 126 males, 404 females, 6 others, 1 prefers not to say	**EE**: TFEQ; **Attachment**: Dimensional attachment using ECR (12-item short form, Lafontaine; global attachment), 9-item Disorganized Attachment scale (global attachment)	Correlation: A-Anx and EE (r=.24 with p<.001). Correlation: A-Avoid and EE (r=.07) (not significant). Correlation: Disorganized attachment and EE (r=.14 with p<.05)
Zakhour et al., 2021, Lebanon [29]	CS; N = 811; Age 18+, M = 27.59 (SD = 11.76); 66.5% females, 33.5% males	**EE**: EES; **Attachment**: Dimensional attachment using SAAM (global attachment); **Mediator**: Body dissatisfaction	Correlation: SAAM-Anx and EE (r = 0.130, p < 0.001). Correlation: SAAM-Sec and with EE (r = -0.148, p < 0.001). Correlation: SAAM-Avoid and EE (r=0.036) (not significant).

Multivariate analysis of covariance (MANCOVA): Individuals with SAAM-Anx have higher EE (Beta = 4.83, p = 0.018).

Multivariate analysis of covariance (MANCOVA): SAAM-Sec and body dissatisfaction together predicted EE (Beta = 3.51, 95% BCa, CI: 0.44 to 6.58, t = 2.25, p = 0.024).

Mediation: Body dissatisfaction mediated between SAAM-Sec and EE, accounting for 12.53% of the effect.

Note: A-Anx= Attachment Anxiety; ARQ= Adolescent Relationship Questionnaire; A-Avoid = Attachment Avoidance; CS = Cross-Sectional; ECR= Experiences in Close Relationships scale; ECR-R: Experiences in Close Relationships scale-Revised; EE = Emotional Eating; EES= Emotional Eating Scale; EES-AF = Angry/Frustrated Eating; EES-Anx = Anxious Eating; EES-Dep = Depressive Eating; EO = Emotional Overeating; EU = Emotional Undereating; IA = Insecure Attachment; LO = Longitudinal; M = Mean; RAAS = The Revised Adult Attachment Scale; RQ= Relationship Questionnaire; SA = Secure Attachment; SAAM = State Attachment Anxiety Model; S-AQS = The Shortened Version of the Attachment Q-Set; SD= Standard Deviation; SES = Socioeconomic Status; SSSP = The Shortened Strange Situation Procedure.

Table S4 Table for qualitative studies

Authors (year, country)	Study design / Sample	Measures & Themes	Results
Mamo & Louka, 2022, Greece [48]	N = 6; Age 18+, M = 22.7 (SD = 3.8); 3 females, 3 males	**EE**: AEBQ;<br><br>**Attachment**: Dimensional attachment using RQ (attachment to significant others);<br><br>**Theme**: Coping with emotions	EO as the activation of an IAS and an alternative way of coping and regulating emotion to support-seeking.<br><br>EU as the transferase of the negative emotions to the body due to the difficulty posed regarding support-seeking.<br><br>The internalized negative view of self, common in individuals with IA, mediated their EE behaviors.<br><br>EU as the reenactment of the occasions when the need of being fed remained unfulfilled and the belief that this need will be met by a significant other.<br><br>EO as the reenactment of the occasions

when the need of being fed was fulfilled and the belief that this need will not be met, so the need for self-reliance in achieving security rises.

EE as a learned behavior stemming from childhood, where food was used as a source of comfort due to lack of emotional support from caregivers.

EE as a learned behavior stemming from childhood, where food was used as a source of comfort due to lack of emotional support from caregivers.

Eating comfort food as a reward for happiness or achievement may reflect a need for attachment-related closeness, which could be the missing element for feeling truly happy.

			Participants often ate childhood comfort foods during distress, suggesting these cues trigger emotional eating for solace.
Hernandez-Hons & Woolley, 2011, USA (Southern California) [49]	N = 8; Age 30–62; Females	**EE**: Not specified;<br><br>**Attachment**: Attachment representations using semi-structured interviews (global attachment, including family, friends, and romantic relationships);<br><br>**Themes**: Personal and cultural foundation;<br><br>Preoccupation with food and eating;<br><br>Relationship history;<br><br>Addiction as a coping mechanism for IA;	EE behaviors were tied to childhood events around food, shaped by attachment relationships and cultural norms.<br><br>Food as a substitute for attachment and a method for evoking positive memories.<br><br>Several participants reported a fixation on food, describing it as "calling to them" and continuing to eat beyond satiety, with this preoccupation increasing as their emotional needs for support and soothing remained unmet.<br><br>Histories of physical, sexual,

Social influences on eating and weight gain;<br><br>Secretive eating;<br><br>EE as reminiscent of ambivalent attachment;<br><br>Emotional hunger.	and psychological abuse with attachment figures led to IA models, persisting into adulthood and resulting in EE as a coping mechanism for failed romantic relationships and lack of support. Participants turned to alcohol, drugs, and EE as unhealthy coping mechanisms to deal with IAs and lack of social support.<br><br>Rejection in seeking emotional closeness led participants to turn to food for comfort.<br><br>Societal judgments about their eating increased secretive eating.<br><br>Participants described secretive eating as both a childhood rebellion and an adult strategy to avoid criticism

		and hide their EE, driven by IA.<br><br>Food became a source of comfort or distraction from pain and stress mirroring ambivalent childhood attachments where parents were both sources of comfort and despair.<br><br>EE as a symptom of IA, a longing for connection, and an attempt to fill emotional voids.
